# Decade-Long Trends in Hospitalization, Outcomes, and Emergency Department Visits for Inflammatory Bowel Diseases in the United States, 2010 to 2020

**DOI:** 10.7759/cureus.77941

**Published:** 2025-01-24

**Authors:** Queeneth Edwards, Oluwatoyin Ayo-Farai, Fidelis E Uwumiro, Babajide Komolafe, Odigili E Chibuzor, Ifeanyi Agu, Henry O Nwuke, Gentle C Uwaoma, Emmanuel S Amadi, Marvis Enyi, Courage Idahor, Chinyere K Omeh

**Affiliations:** 1 Internal Medicine, Georgia Southern University, Statesboro, Georgia, USA; 2 Epidemiology and Public Health, Jiann-Ping Hsu College of Public Health, Georgia Southern University, Statesboro, USA; 3 Internal Medicine, Prime Healthcare-SRGA, Riverdale, USA; 4 Internal Medicine, College of Medicine, University of Lagos, Lagos, NGA; 5 Public Health, University of Wisconson Milwaukee, Milwaukee, USA; 6 Internal Medicine, Imo State University College of Medicine, Owerri, NGA; 7 Public Health, University of Chester, Chester, GBR; 8 Internal Medicine, College of Medicine, University of Nigeria, Enugu, NGA; 9 Internal Medicine, Hallel Hospital Port Harcourt, Port Harcourt, NGA; 10 Internal Medicine, Imo State University Teaching Hospital, Owerri, NGA; 11 Emergency Medicine, Barking, Havering and Redbridge University Hospitals NHS Trust, London, GBR; 12 Internal Medicine, 161 Nigerian Airforce Hospital, Makurdi, NGA

**Keywords:** crohn disease, emergency department utilizaiton, inflammatory bowel diseases, joinpoint regression, large bowel resection, small bowel resection, ulcerative colitis

## Abstract

Background

Data on trends in inflammatory bowel disease (IBD) hospitalizations in the literature are sparse and conflicting. This study evaluated trends in hospitalization and emergency department (ED) visits for IBD between 2010 and 2020 using large data from the United States national inpatient and emergency department sample databases.

Methods

We employed joinpoint regression analysis and Cuzick’s tests to examine trends in hospitalizations, emergency department (ED) visits, and outcomes of hospitalization for IBD using nationwide inpatient and ED sample databases. Hospitalization costs were adjusted for inflation using the medical expenditure panel survey index.

Results

We analyzed 2,504,288 Crohn's Disease (CD) and 1,367,809 ulcerative colitis (UC) hospitalizations. There was an uptrend in the mean age of patients with IBD from 52.3 years in 2010 to 55.8 years in 2020 (P <0.001). Hospitalizations for IBD showed an upward trend with an average annual percent change (APC) of 0.92% (confidence interval [CI]: 0.67-1.17; P<0.001) and a marked increase in CD hospitalization until 2014 (APC, 2.16%; CI, 1.35-4.64; P=0.040). After 2014, CD hospitalizations showed a downward trend to 219,200 with an AAPC of -0.1% (CI: -1.79 to 1.61; P=0.890), whereas UC hospitalizations steadily increased over the decade (120,346 to 122,485; APC, 0.63%; CI, 0.52-0.74; P<0.001). Mortality rates increased by an average APC of 3.16% (P=0.002), especially among the middle-aged and older adults. Aggregate annual IBD hospitalization costs were $9.1 billion higher in 2020 than in 2010 (APC: 3.97% (CI: 2.98-4.97; P<0.001). There were 6,243,807 ED visits for IBDs over the study period. There was no significant change in the overall number of ED visits for IBD over the study period (574,038 to 448,647; APC: 0.1%; CI: -0.42 to 0.54; P=0.792). There was an uptrend in the total number of in-hospital procedures for IBD (622,647 to 642,210; APC: 0.64%; CI: 0.35-0.93; P=0.001). There was an uptrend in the incidence of combined incidences of malnutrition, anemia, bowel perforations, fistulae, and critical care admission for IBD (P*_trend_* for all < 0.001).

Conclusion

IBD hospitalization rates have increased with aging patient demographics, rising mortality rates, and increased healthcare spending over the past decade.

## Introduction

Inflammatory bowel disease (IBD), including Crohn's disease (CD) and ulcerative colitis (UC), represents a group of chronic, idiopathic inflammatory disorders of the gastrointestinal tract. The prevalence of IBD has been on the rise globally [1,2] with significant variations in hospitalization across different regions and populations [3,4]. In the United States, IBD poses a substantial burden on patients’ quality of life and incurs considerable healthcare use and costs, primarily due to hospitalizations and emergency department (ED) visits [5,6].

Over the last decade, the management of Inflammatory Bowel Disease (IBD) has improved significantly due to advancements in diagnostic methods, a deeper understanding of disease mechanisms, and the introduction of new treatments, including biologics [7] and small molecules [8]. These developments have markedly influenced IBD management, potentially impacting hospitalization and emergency department visit trends for affected patients [9-11].

We examined trends in demographics, hospitalizations, emergency department (ED) visits, and clinical outcomes for inflammatory bowel disease (IBD) in the United States from 2010 to 2020. This decade-long analysis offers a comprehensive overview of the evolving patterns in IBD-related healthcare utilization. Our findings shed light on recent trends, laying the groundwork for future research and intervention. By examining these trends, the study aims to guide healthcare providers and researchers in understanding the disease burden and patient demographics, establishing a benchmark to evaluate progress made over the past decade in addressing this complex and challenging condition.

## Materials and methods

Data sources and variables

The Healthcare Cost and Utilization Project (HCUP, pronounced "H-Cup") is a suite of healthcare databases and related software tools and products developed through a Federal-State-Industry partnership and sponsored by the Agency for Healthcare Research and Quality (AHRQ). HCUP databases are among the largest and most comprehensive sources of hospital care data in the United States, encompassing all payers and the uninsured. These databases facilitate research on hospital utilization, access, charges, quality, and outcomes [12]. Among its several databases, the Nationwide Inpatient Sample (NIS) and the Nationwide Emergency Department Sample (NEDS) are particularly noteworthy for their scope and utility in inpatient and ED research. The NIS is the largest publicly available all-payer inpatient care database in the United States [12,13]. It contains data from approximately 8 million hospital stays each year across approximately 1000 hospitals, which are sampled to approximate a 20% stratified sample of U.S. community hospitals. The NIS is designed to provide national estimates of hospital inpatient stays and to enable analyses of trends over time. The database includes clinical and resource use information typically available from discharge abstracts. It also allows for the study of various health policy issues, including the cost and quality of health services, medical practice patterns, access to health care programs, and outcomes of treatments at the national, state, and local market levels. Mortality variables within these databases typically include in-hospital death rates, which capture whether a patient died during their hospital stay. This crucial data point allows for the analysis of patient outcomes and the effectiveness of hospital care. In addition, HCUP databases detail extensive resource use variables, such as the length of hospital stay, number of procedures, total charges, and costs associated with each visit or hospitalization. We used data from the NIS to analyze national trends in hospitalization, healthcare resource utilization, and IBD outcomes between 2010 and 2020.

The NEDS is the largest all-payer emergency department (ED) database in the United States. It is designed to enable analyses of ED utilization patterns and support public health professionals, administrators, policymakers, and clinicians in their decision-making processes. It contains data from more than 30 million ED visits each year from a sample of approximately 950 hospitals, approximating a 20% stratified sample of U.S. hospital-owned EDs. NEDS captures information on patient demographics, hospital characteristics, ED visit details, diagnoses and procedures, sources of payment, and charges for ED services. NEDS is particularly valuable for examining the national rates of ED usage, identifying patterns of care for common and emerging health conditions, and assessing the impact of policy changes on ED practices. It also facilitates research on a variety of issues, such as the reasons for ED visits, outcomes of ED visits (including hospital admission rates), quality of emergency care, and discharge disposition from the ED (indicating what kind of discharge occurred for each ED visit).

Both the NIS and NEDS databases provide a comprehensive and detailed view of hospital-based care in the United States. Thus, we used both databases to gain insights into ED use and hospitalization trends for IBD over the study period. By offering a granular look at hospital inpatient and ED care across the nation, the NIS and NEDS databases help identify disparities in healthcare access and outcomes, evaluate the efficiency of healthcare delivery, and assess the impact of healthcare reforms. For instance, analyses of NIS data can reveal trends in hospital readmissions for chronic diseases, whereas NEDS data can provide insights into the prevalence of nonurgent visits to the ED and the factors driving such patterns.

Study selection and statistical analysis

Our analysis was conducted using a comprehensive, multistep statistical approach using Stata statistical software (StataCorp LLC, College Station, TX, USA) for data analysis and the Joinpoint Regression Program version 5.0 (National Cancer Institute, Bethesda, USA) for trend analysis.

Joinpoint regression analysis is a statistical method used to identify points where a significant change occurs in the trend of data over time. This technique is particularly useful in epidemiology and health research for analyzing trends in disease incidence, mortality rate, and other health-related events. By fitting a series of joined straight lines to the data points, joinpoint regression can be used to pinpoint where the rate of increase or decrease significantly shifts. These points, known as "joinpoints," indicate periods when public health interventions were introduced, changes in diagnostic criteria occurred, or other external factors influenced the trend. This method makes it possible to describe and understand complex trend patterns more accurately, facilitating a deeper understanding of the temporal dynamics of the data under study. Therefore, joinpoint regression is a powerful tool for detecting and quantifying changes in trends over time, providing valuable guidance for policy-making and resource allocation in healthcare planning and intervention strategies. Joinpoint regression analysis summarizes data by segmenting it into linear phases delineated by joinpoints, where there is a significant change in trend. For each phase, the annual percentage change (APC) is calculated to quantify the rate of change, and the average annual percentage change (AAPC) often summarizes the overall trend across the study period. The analysis determines the optimal number of joinpoints through statistical testing and provides a detailed view of the trend changes over time. Confidence intervals (CI) for APCs and AAPCs indicate statistical uncertainties. The results, including the identified joinpoints and APCs for each segment, are typically presented graphically, showcasing the trend changes and providing a clear and comprehensive summary of the data's behavior over the observed period.

Data were extracted from the NIS and NEDS and included all adult admissions with a primary diagnosis of IBD, encompassing both CD and UC, identified by specific ICD-10 codes (K50 and K51). Initially, descriptive statistics were used to summarize the demographic characteristics of the study population, alongside clinical variables such as disease subtype (CD or UC), comorbidity burden (using the Charlson Comorbidity Index), and hospital characteristics (hospital location and teaching status). Continuous variables are presented as means ± standard deviations (SD) or medians with interquartile ranges (IQR) depending on their distribution, as assessed by the Shapiro-Wilk test. Categorical variables are summarized as percentages, whereas hospitalization and ED visits are presented as absolute counts. APCs or AAPCs in hospitalization and ED visit rates were calculated, with statistical significance set at P < 0.05. This method allowed for the detection of shifts in admission rates and the assessment of whether trends were increasing, stable, or decreasing over the decade. Comparisons between hospitalizations and ED visits for CD versus UC were performed using the chi-square test for categorical variables and the student’s t-test or Mann-Whitney U test for continuous variables, as appropriate. Hierarchical multivariable logistic regression was utilized to identify biodemographic variables significantly correlated with mortality during the study period. Results of multivariable regression are presented as adjusted odds ratio (aOR) with 95% confidence interval (CI).

We analyzed resource utilization by calculating the total and average hospital charges, procedure counts, and lengths of hospitalization and ED visits. Trends in in-hospital IBD complications and resource use were examined using Cuzick’s trend test, with inflation adjustments made using the medical expenditure panel survey index for consistent dollar values throughout the study.

Ethical considerations

This study was conducted using deidentified public NIS and NEDS data from the HCUP databases, adhering to all applicable AHRQ guidelines and regulations. Given the deidentified nature of the data, Institutional Review Board approval was not required.

## Results

Trends in IBD hospitalizations and biodemographic characteristics

Of the 339 million hospitalizations included in our study, there were 3,872,097 hospitalizations for IBD, with 2,504,288 being for Crohn’s disease and 1,367,809 being for ulcerative colitis. The mean age for all IBD hospitalizations was 52.9 ± 20.3. More females (57.6%) than males (42.4%) and more White Americans (79.5%) than Black (10.4%), Hispanic (6.2%), or Asian (1.2%) were hospitalized during the study period.

Trends in the biodemographic data of hospitalizations with IBD are summarized in Table 1.

**Table 1:** Prevalence and Trends in Biodemographic Characteristics of Hospitalizations for Inflammatory Bowel Disease The data is presented as frequency (n) with percentages (%) for categorical data and as means with standard deviation for continuous CCI, Charlson comorbidity index; IBD, inflammatory bowel disease; SD, standard deviation; CD, Chron's disease; UC, ulcerative colitis P_trend_ = Cuzick’s non-parametric trend test; significance level set at P_trend_ <0.05.

Variable	2010 (N = 332,802), n (%) Unless Otherwise Specified	2011 (N = 341,803), n (%) Unless Otherwise Specified	2012 (N = 343,790), n (%) Unless Otherwise Specified	2013 (N = 355,050), n (%) Unless Otherwise Specified	2014 (N = 357,898), n (%) Unless Otherwise Specified	2015 (N = 357,703), n (%) Unless Otherwise Specified	2016 (N = 358,945), n (%) Unless Otherwise Specified	2017 (N = 359,350), n (%) Unless Otherwise Specified	2018 (N = 362,230), n (%) Unless Otherwise Specified	2019 (N = 360,841), n (%) Unless Otherwise Specified	2020 (N = 341,685), n (%) Unless Otherwise Specified	P_trend_
Hospitalizations for CD	212,456 (63.8)	220,014 (64.4)	220,748 (64.2)	231,490 (65.1)	233,893 (65.4)	232,651 (65.0)	234,260 (65.3)	233,630 (65.0)	234,590 (64.8)	231,356 (64.1)	219,200 (64.2)	<0.001
Hospitalizations for UC	120,346 (36.2)	121,789 (35.6)	123,042 (35.8)	123,560 (34.8)	124,005 (34.6)	125,052 (35.0)	124,685 (34.7)	125,720 (35.0)	127,640 (35.2)	129,485 (35.9)	122,485 (35.8)	<0.001
Mean age ± SD, years	52.3 ± 19.8	52.0 ± 20.2	53.6 ± 18.9	51.7 ± 19.4	52.1 ± 20.2	52.3 ± 20.8	51.9 ± 18.8	52.3 ± 18.0	52.8 ± 20.2	54.5 ± 19.5	55.8 ± 18.3	0.001
Age categories												0.031
Young adults (18-44 years)	105,165 (31.6)	111,770 (32.7)	118,951 (34.6)	119,652 (33.7)	120,254 (33.6)	116,969 (32.7)	120,247 (33.5)	118,226 (32.9)	115,189 (31.8)	117,273 (32.5)	110,706 (32.4)	
Middle-aged (45-64 years)	101,172 (30.4)	106,643 (31.2)	114,482 (33.3)	119,297 (33.6)	117,033 (32.7)	112,676 (31.5)	111,632 (31.1)	111,039 (30.9)	110,842 (30.6)	107,891 (29.9)	104,556 (30.6)	
Elderly (≥65 years)	126,465 (38.0)	123,391 (36.1)	103,481 (30.1)	116,101 (32.7)	120,612 (33.7)	128,415 (35.9)	127,067 (35.4)	130,085 (36.2)	136,198 (37.6)	135,676 (37.6)	126,423 (37.0)	
Female	195,355 (58.7)	196,879 (57.6)	205,586 (59.8)	212,675 (59.9)	219,391 (61.3)	215,337 (60.2)	213,572 (59.5)	215,251 (59.9)	217,700 (60.1)	215,422 (59.7)	204,328 (59.8)	
Race												<0.001
White	262,581 (78.9)	270,708 (79.2)	266,781 (77.6)	282,620 (79.6)	283,455 (79.2)	281,512 (78.7)	286,438 (79.8)	285,324 (79.4)	287,973 (79.5)	286,869 (79.5)	270,956 (79.3)	
Black	37,274 (11.2)	36,231 (10.6)	41,255 (12.0)	35,860 (10.1)	37,221 (10.4)	37,917 (10.6)	37,689 (10.5)	37,732 (10.5)	36,947 (10.2)	37,527 (10.4)	35,877 (10.5)	
Hispanic	37,274 (6.3)	18,799 (5.5)	21,659 (6.3)	21,658 (6.1)	22,190 (6.2)	23,608 (6.6)	21,178 (5.9)	22,280 (6.2)	23,907 (6.6)	22,372 (6.2)	21,526 (6.3)	
Asian/Pacific Islander	3,328 (1.0)	5,811 (1.7)	4,813 (1.4)	4,971 (1.4)	4,295 (1.2)	6,439 (1.8)	3,948 (1.1)	3,953 (1.1)	4,347 (1.2)	4,330 (1.2)	4,100 (1.2)	
Native American	998 (0.3)	1,709 (0.5)	2,407 (0.7)	1,420 (0.4)	2,147 (0.6)	1,073 (0.3)	1,436 (0.4)	1,437 (0.4)	1,449 (0.4)	1,443 (0.4)	1,367 (0.4)	
Others	7,655 (2.3)	8,203 (2.4)	7,220 (2.1)	8,521 (2.4)	8,590 (2.4)	7,154 (2.0)	8,256 (2.3)	8,624 (2.4)	8,331 (2.3)	8,299 (2.3)	7,859 (2.3)	
CCI score												<0.001
0	146,433 (44.0)	138,088 (40.4)	156,424 (45.5)	153,027 (43.1)	164,991 (46.1)	160,251 (44.8)	163,679 (45.6)	156,317 (43.5)	152,137 (42.0)	153,718 (42.6)	133,599 (39.1)	
1	68,557 (20.6)	72,804 (21.3)	69,446 (20.2)	73,850 (20.8)	76,232 (21.3)	75,118 (21.0)	73,225 (20.4)	74,026 (20.6)	74,257 (20.5)	73,972 (20.5)	69,704 (20.4)	
2	36,608 (11.0)	42,042 (12.3)	38,848 (11.3)	44,026 (12.4)	45,453 (12.7)	46,144 (12.9)	45,586 (12.7)	45,637 (12.7)	47,814 (13.2)	46,909 (13.0)	46,127 (13.5)	
≥3	80,538 (24.2)	88,869 (26.0)	79,416 (23.1)	84,147 (23.7)	71,222 (19.9)	76,191 (21.3)	76,814 (21.4)	83,369 (23.2)	87,660 (24.2)	86,241 (23.9)	95,330 (27.9)	
Hospital bed size												0.057
Small	56,576 (17.0)	64,943 (19.0)	70,821 (20.6)	73,140 (20.6)	78,380 (21.9)	70,110 (19.6)	63,892 (17.8)	68,995 (19.2)	73,170 (20.2)	71,447 (19.8)	74,829 (21.9)	
Medium	94,183 (28.3)	94,338 (27.6)	94,198 (27.4)	97,284 (27.4)	96,633 (27.0)	98,726 (27.6)	100,505 (28.0)	100,259 (27.9)	100,338 (27.7)	99,592 (27.6)	91,913 (26.9)	
Large	182,043 (54.7)	182,181 (53.3)	178,771 (52.0)	181,431 (51.1)	182,886 (51.1)	188,867 (52.8)	194,548 (54.2)	190,096 (52.9)	188,722 (52.1)	189,802 (52.6)	174,943 (51.2)	
Hospital region												0.825
Northeast	71,552 (21.5)	73,488 (21.5)	72,883 (21.2)	74,916 (21.1)	79,453 (22.2)	77,979 (21.8)	77,891 (21.7)	76,900 (21.4)	85,124 (23.5)	80,107 (22.2)	74,829 (21.9)	
Midwest	85,863 (25.8)	87,160 (25.5)	90,417 (26.3)	89,473 (25.2)	88,401 (24.7)	89,068 (24.9)	90,813 (25.3)	87,322 (24.3)	82,226 (22.7)	85,880 (23.8)	77,904 (22.8)	
South	121,473 (36.5)	124,417 (36.4)	123,421 (35.9)	132,079 (37.2)	127,412 (35.6)	127,700 (35.7)	126,708 (35.3)	131,881 (36.7)	122,434 (33.8)	123,408 (34.2)	116,856 (34.2)	
West	54,247 (16.3)	56,397 (16.5)	57,069 (16.6)	58,583 (16.5)	62,632 (17.5)	63,313 (17.7)	63,892 (17.8)	63,605 (17.7)	72,446 (20.0)	71,447 (19.8)	67,654 (19.8)	
Median household income												0.283
0–25th percentile	73,882 (22.2)	76,564 (22.4)	72,540 (21.1)	79,531 (22.4)	89,475 (25.0)	87,280 (24.4)	86,506 (24.1)	85,166 (23.7)	82,951 (22.9)	85,880 (23.8)	80,638 (23.6)	
26th–50th percentile	79,873 (24.0)	85,793 (25.1)	87,666 (25.5)	91,603 (25.8)	89,117 (24.9)	92,645 (25.9)	88,300 (24.6)	91,994 (25.6)	94,542 (26.1)	95,623 (26.5)	87,813 (25.7)	
51st-75th percentile	89,191 (26.8)	89,552 (26.2)	92,480 (26.9)	89,473 (25.2)	93,411 (26.1)	92,287 (25.8)	94,403 (26.3)	93,431 (26.0)	96,353 (26.6)	89,849 (24.9)	88,496 (25.9)	
75-100th percentile	90,189 (27.1)	90,236 (26.4)	91,448 (26.6)	94,798 (26.7)	86,253 (24.1)	85,491 (23.9)	90,095 (25.1)	89,119 (24.8)	88,746 (24.5)	89,128 (24.7)	84,738 (24.8)	
Location/teaching status of the hospital												0.004
Rural	25,959 (7.8)	25,293 (7.4)	25,097 (7.3)	26,274 (7.4)	22,905 (6.4)	23,251 (6.5)	22,614 (6.3)	22,280 (6.2)	25,356 (7.0)	25,620 (7.1)	23,918 (7.0)	
Urban nonteaching	76,877 (23.1)	70,753 (20.7)	62,226 (18.1)	56,098 (15.8)	82,674 (23.1)	72,971 (20.4)	66,046 (18.4)	57,855 (16.1)	65,926 (18.2)	70,364 (19.5)	54,328 (15.9)	
Urban teaching	229,966 (69.1)	245,756 (71.9)	256,467 (74.6)	272,678 (76.8)	252,318 (70.5)	261,481 (73.1)	269,927 (75.2)	279,215 (77.7)	270,948 (74.8)	264,857 (73.4)	263,439 (77.1)	

There was an upward trend in the mean age for IBD hospitalizations from 52.3 ± 19.8 years in 2010 to 55.8 ± 18.3 years in 2020 (P < 0.001) and in hospitalizations among females (58.7% in 2010 to 59.8% in 2020). The proportion of patients with a CCI score of ≥3 showed an upward trend over the study period (from 24.2% in 2010 to 27.9% in 2020; P < 0.001) indicating a trend towards greater comorbidity burden over the period. An upward trend was observed in the proportion of patients hospitalized at smaller hospitals (17.0% in 2010 to 21.9% in 2020; P < 0.001). Hospitalizations for individuals in the 0-25th and 26th-50th percentiles also showed an upward trend (22.2% to 23.6% and 24% to 25.7% respectively; P for all < 0.001).

**Figure 1:**
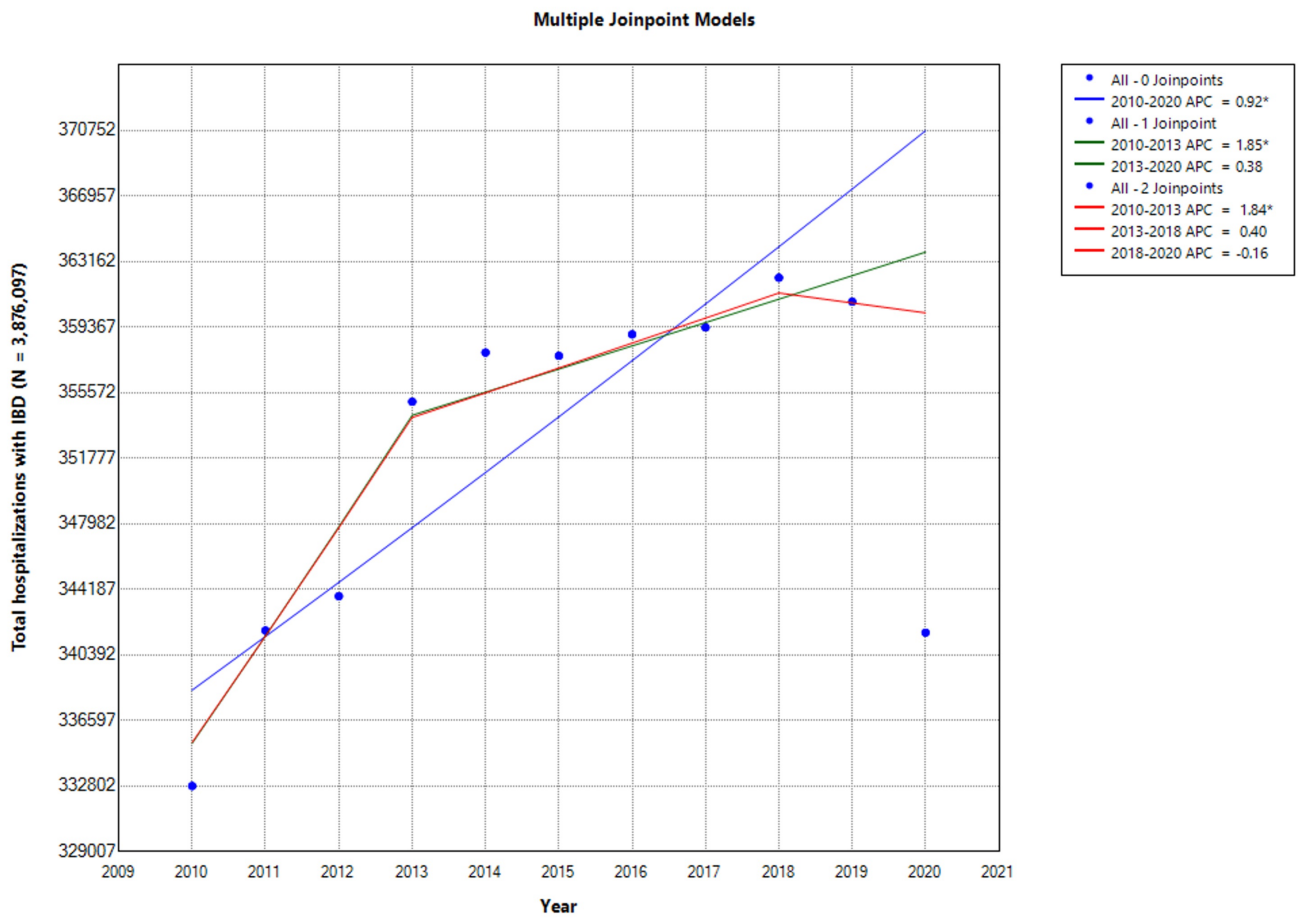
Inflammatory bowel disease hospitalization trends * Indicates that the annual percentage change (APC) is significantly different from zero at the alpha level = 0.05. Negative values indicate a downward trend. In Joinpoint software, different colors distinguish linear trend segments between joinpoints, highlighting shifts in direction or magnitude and aiding in visualizing statistically significant changes.

Hospitalizations for IBD showed an upward trend over the study period with an AAPC of 0.92% (CI: 0.67-1.17; P < 0.001; Figure 1).

**Figure 2:**
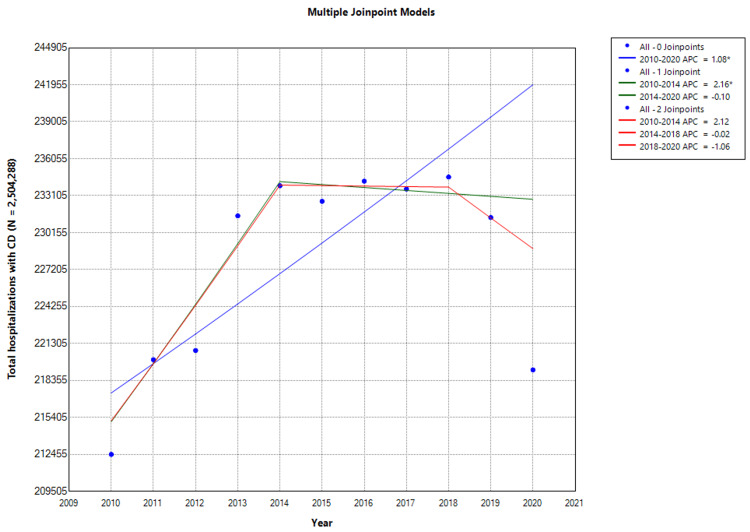
Trends in Total Crohn’s Disease Hospitalizations * Indicates that the Annual Percent Change (APC) is significantly different than zero at the alpha level = 0.05. Negative values indicate a downward trend. CD = Crohn disease The proportion of CD hospitalizations is the percentage of inflammatory bowel disease (IBD) hospitalizations attributed to CD. In Joinpoint software, different colors distinguish linear trend segments between joinpoints, highlighting shifts in direction or magnitude and aiding in visualizing statistically significant changes.

**Figure 3:**
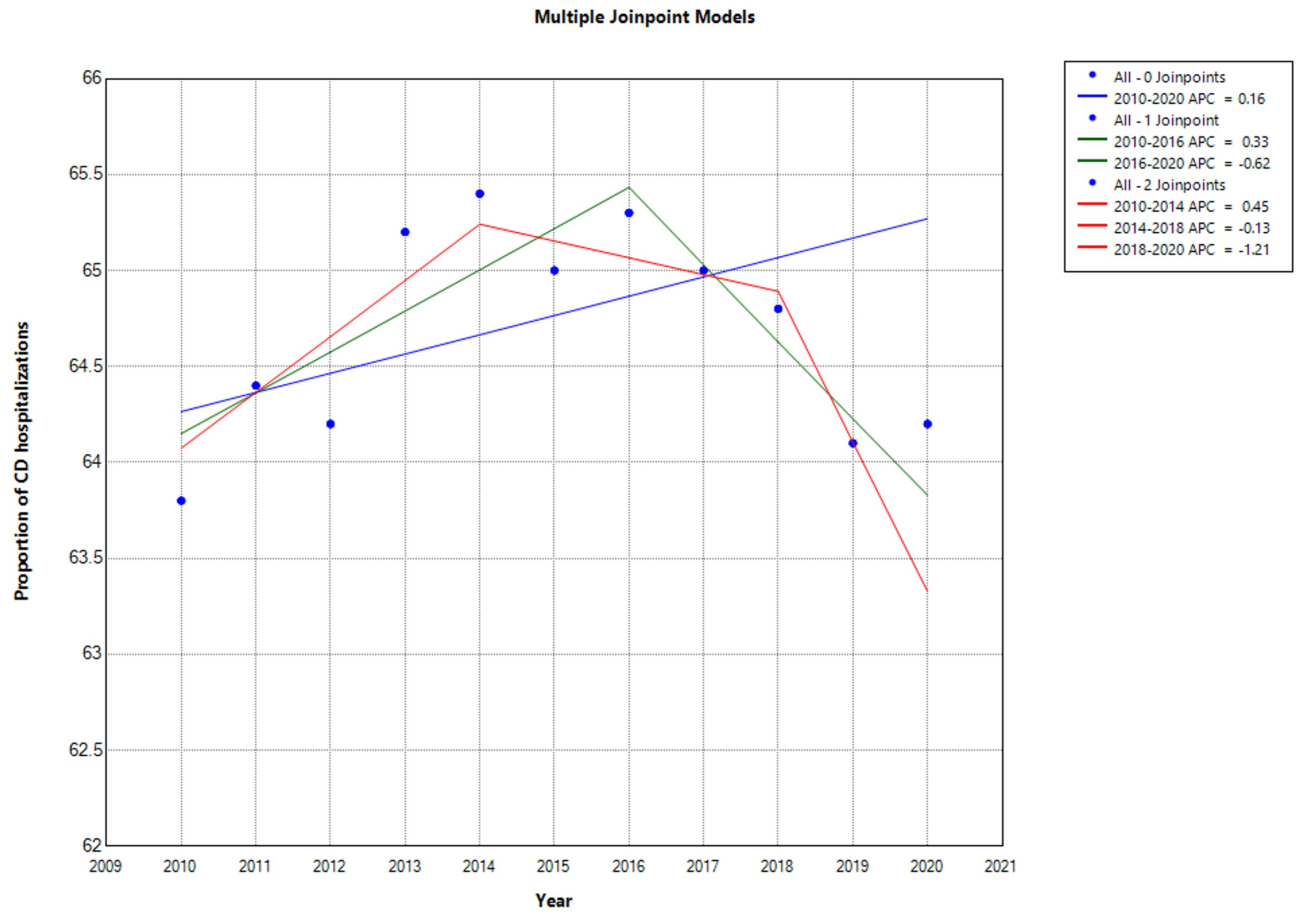
Trend in Proportion of Inflammatory Bowel Disease Attributable to Crohn’s Disease * Indicates that the Annual Percent Change (APC) is significantly different than zero at the alpha level = 0.05. Negative P-values indicate a downward trend. CD = Crohn's disease The proportion of CD hospitalizations is the annual percentage of IBD hospitalizations attributed to CD. In Joinpoint software, different colors distinguish linear trend segments between joinpoints, highlighting shifts in direction or magnitude and aiding in visualizing statistically significant changes.

Total hospitalizations for CD showed an upward trend with an AAPC of 1.08% (CI: 0.72-1.44: P < 0.001). This uptrend was particularly marked between 2010 and 2014, increasing from 212,456 to 233,893 with an APC of 2.16% (CI: 1.35 to 4.64; P = 0.04). After 2014, CD hospitalizations showed a downward trend to 219,200 with an AAPC of -0.1% (CI: -1.79 to 1.61; P = 0.890; Figure 2 and Figure 3). However, this was not statistically significant (Figure 2). A marked reduction (6.3%) in CD hospitalizations was observed between 2014 and 2020 (P = 0.03).

**Figure 4:**
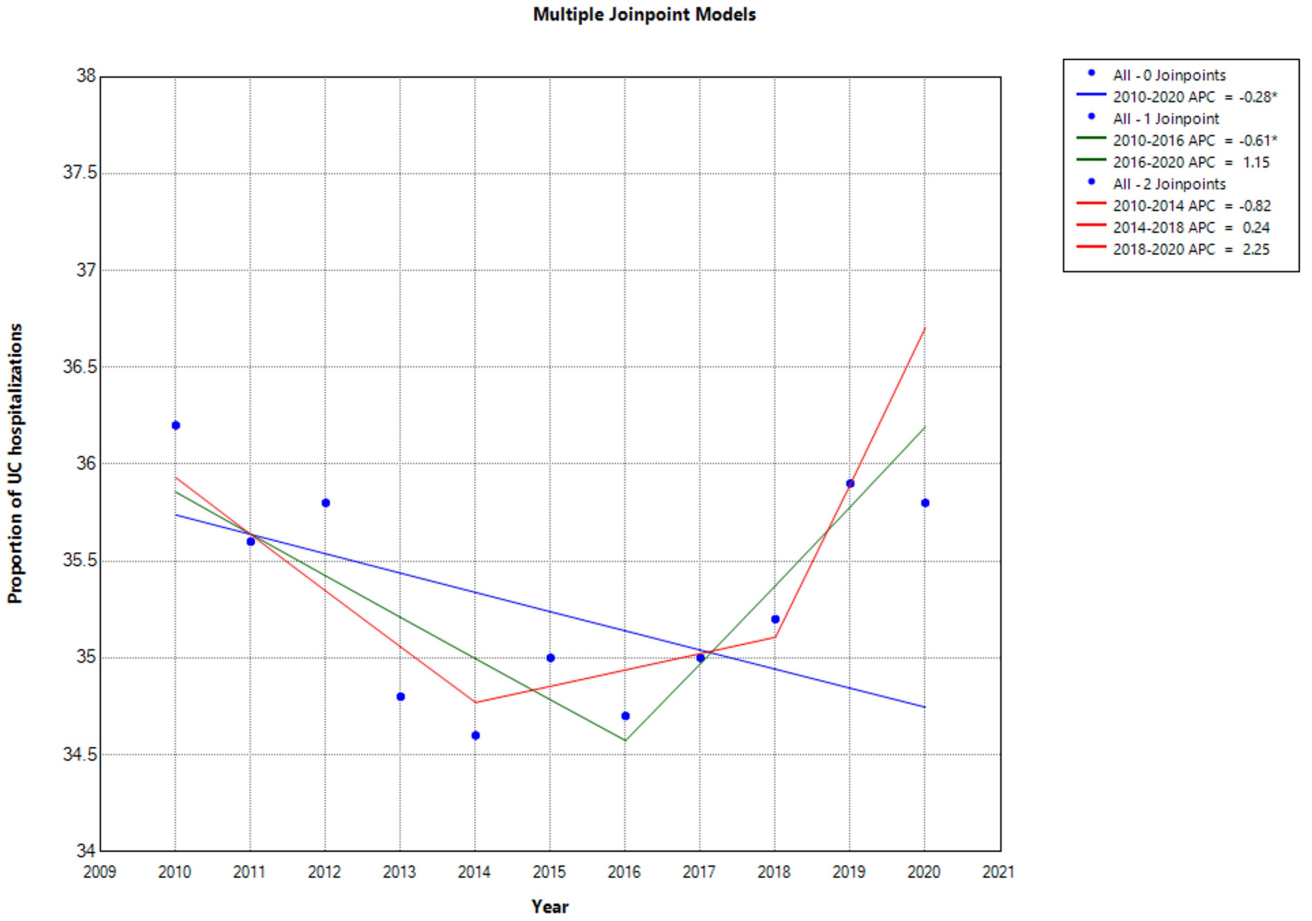
Trend in Proportion of Inflammatory Bowel Disease Attributable to Ulcerative Colitis * Indicates that the Annual Percent Change (APC) is significantly different than zero at the alpha level = 0.05. Negative P-values indicate a downward trend. UC = Ulcerative colitis The proportion of UC hospitalizations is the annual percentage of inflammatory bowel disease (IBD) hospitalizations attributed to ulcerative colitis. In Joinpoint software, different colors distinguish linear trend segments between joinpoints, highlighting shifts in direction or magnitude and aiding in visualizing statistically significant changes.

The proportion of IBD hospitalizations due to UC showed a downward trend from 39.0% of all IBD hospitalizations in 2010 to 33% in 2014 (APC: -3.9%; CI: -4.9% to -2.81; P < 0.001), and then an uptrend from 33% in 2014 to 36% in 2020 (APC: 1.9%; CI: 0.3%-3.55%; P = 0.027; Figure 4).

Outcomes

Mortality

The unadjusted outcomes of IBD hospitalizations are presented in Table 2. There was a 3.16% average annual percent increase in mortality (CI: 1.50-4.83; P = 0.002) over the study period when adjusted for age, sex, and race. Compared with young adults, middle-aged and older patients had adjusted odds ratios (aOR) of 2.66 (95% CI: 1.27- 5.55, P = 0.009) and 2.73 (95% CI: 1.27-5.86, P = 0.010), respectively, for mortality. Female sex was associated with lower adjusted odds of mortality (aOR: 0.78; 95% CI: 0.74-0.83; P < 0.001). The mortality risk was higher among native Americans (aOR: 1.68; 95% CI: 1.06-2.66; P = 0.028). There was no significant difference in adjusted mortality among Blacks (P = 0.754) or Hispanics (P = 0.369) compared with white Americans.

**Table 2:** Trends in Mortality Rate for IBD Hospitalizations CCI, Charlson comorbidity index; IBD, inflammatory bowel disease; SD, standard deviation; CD, Crohn's disease; UC, ulcerative colitis; ED, emergency department P_trend_ = Cuzick's non-parametric trend test; significance level set at P_trend_<0.05.

Variable	2010, n (%)	2011, n (%)	2012, n (%)	2013, n (%)	2014, n (%)	2015, n (%)	2016, n (%)	2017, n (%)	2018, n (%)	2019, n (%)	2020, n (%)	P_trend_
Hospitalizations for IBD	4,326 (1.3)	4,785 (1.4)	4,813 (1.4)	5,326 (1.5)	5,726 (1.6)	5,723 (1.6)	5,384 (1.5)	5,390 (1.5)	5,071 (1.4)	7,217 (2.0)	5,467 (1.6)	<0.001
Hospitalizations for CD	2,762 (1.3)	3,080 (1.4)	3,135 (1.42)	3,704 (1.6)	3,275 (1.4)	3.024 (1.3)	3,045 (1.3)	3,084 (1.32)	2,815 (1.2)	3,933 (1.7)	3,946 (1.8)	<0.001
Hospitalizations for UC	1,685 (1.4)	1,583 (1.3)	1,846 (1.5)	1,730 (1.4)	2,108 (1.7)	2,126 (1.7)	2,244 (1.8)	2,389 (1.9)	2,489 (1.95)	3,289 (2.54)	2,450 (2.0)	0.001
Males	6,323 (1.9)	6,152 (1.8)	7,220 (2.1)	9,941 (2.8)	7,874 (2.2)	5,008 (1.4)	5,743 (1.6)	5,750 (1.6)	6,158 (1.7)	6,495 (1.8)	8,200 (2.4)	0.001
Females	6,323 (1.9)	6,152 (1.8)	7,907 (2.3)	6,746 (1.9)	7,158 (2.0)	6,796 (1.9)	5,025 (1.4)	5,390 (1.5)	4,709 (1.3)	5,773 (1.6)	6,150 (1.8)	<0.001
Age categories												
18-40 years	8,986 (2.7)	9,229 (2.7)	7,907 (2.3)	8,876 (2.5)	10,737 (3.0)	10,016 (2.8)	11,127 (3.1)	11,140 (3.1)	10,142 (2.8)	11,186 (3.1)	13,326 (3.9)	<0.001
41–60 years	5,990 (1.8)	5,811 (1.7)	5,844 (1.7)	6,391 (1.8)	7,516 (2.1)	9,300 (2.6)	8,256 (2.3)	6,828 (1.9)	10,867 (3.0)	11,547 (3.2)	8,542 (2.5)	<0.001
>60 years	8,653 (2.6)	9,571 (2.8)	10,658 (3.1)	11,007 (3.1)	9,663 (2.7)	10,731 (3.0)	11,845 (3.3)	11,140 (3.1)	10,142 (2.8)	11,547 (3.2)	12,984 (3.8)	<0.001
Race												
White	5,990 (1.8)	6,494 (1.9)	6,876 (2.0)	9,231 (2.6)	6,442 (1.8)	7,512 (2.1)	5,384 (1.5)	5,750 (1.6)	5,433 (1.5)	6,495 (1.8)	7,175 (2.1)	<0.001
Black	6,656 (2.0)	7,520 (2.2)	5,844 (1.7)	7,456 (2.1)	9,305 (2.6)	5,008 (1.4)	4,307 (1.2)	4,672 (1.3)	3,985 (1.1)	4,691 (1.3)	6,150 (1.8)	0.003
Hispanic	5,990 (1.8)	4,102 (1.2)	5,501 (1.6)	6,746 (1.9)	4,295 (1.2)	5,723 (1.6)	5,743 (1.6)	3,234 (0.9)	4,347 (1.2)	5,052 (1.4)	6,150 (1.8)	0.007
Asian/Pacific Islander	4,992 (1.5)	7,520 (2.2)	9,626 (2.8)	3,906 (1.1)	6,442 (1.8)	7,512 (2.1)	4,307 (1.2)	6,109 (1.7)	6,882 (1.9)	6,134 (1.7)	7,175 (2.1)	0.547
Native American	6,989 (2.1)	5,811 (1.7)	16,158 (4.7)	7,811 (2.2)	7,158 (2.0)	4,650 (1.3)	7,179 (2.0)	8,984 (2.5)	4,347 (1.2)	6,856 (1.9)	10,592 (3.1)	0.563
Other races	6,989 (2.1)	8,887 (2.6)	7,563 (2.2)	4,616 (1.3)	7,516 (2.1)	8,585 (2.4)	4,666 (1.3)	7,906 (2.2)	4,709 (1.3)	5,052 (1.4)	4,442 (1.3)	0.034

An uptrend in mortality was observed among men hospitalized with IBD between 2018 and 2020 with an APC of 31.48% (CI: 0.92-71.28; P = 0.046).

Number of Procedures and Hospitalization Costs

There was an uptrend in the total number of in-hospital procedures from 622,647 in 2010 to 642,210 in 2020 (APC: 0.64%; CI: 0.35-0.93; p = 0.001). There was no significant difference in the adjusted mean number of procedures performed between males and females (p = 0.745; Table 3).

**Table 3:** Total Number of Procedures The number of procedures is presented as frequencies for each year from 2010 to 2020 P_trend_ = Cuzick's nonparametric trend test; significance set at P_trend_<0.05

Variable	Frequencies (n)											
	2010	2011	2012	2013	2014	2015	2016	2017	2018	2019	2020	Ptrend
All IBD hospitalizations	602,647	604,265	606,834	608,784	610,464	611,735	613,800	621,330	635,350	636,484	642,210	<0.001
Males	391,817	391,875	393,110	393,045	393,481	393,371	393,845	396,585	407,215	406,638	416,100	0.001
Females	210,830	212,390	213,724	215,739	216,983	218,364	219,955	224,745	228,135	229,846	226,110	<0.001
18-40 years	190,936	191,354	191,736	192,021	192,679	193,674	193,100	188,720	190,045	191,749	190,580	<0.001
41–60 years	186,936	188,354	189,836	191,023	192,637	193,825	194,745	197,200	197,300	196,394	195,300	<0.001
>60 years	224,775	224,557	225,262	225,740	225,148	224,236	225,955	235,419	248,005	248,341	256,330	<0.001

There was a $3,286 increase in mean hospital costs (THC) over the study period (P<0.001) after adjusting for age, sex, race, illness severity, and comorbidity index. Overall, adjusted hospitalization costs were $9.1 billion higher in 2020 than in 2010 (P < 0.001; Table 3). Black, Hispanic, and Asian/Pacific Islanders had increases in adjusted THC of $2,250 (P = 0.002), $13,911 (P < 0.001), and $21,854 (P < 0.001) when compared with White Americans. Overall hospitalization costs increased by an APC of 3.97% (CI: 2.98-4.97; P < 0.001). A similar trend was observed among females (APC: 6.04%; CI: 5.05-9.10; P < 0.001), middle-aged (APC: 5.87%; CI: 5.57-6.83; P < 0.001), and elderly patients with IBD (APC: 6.52%; CI: 5.07-9.35; P < 0.001; Table 4).

**Table 4:** Total Hospitalization Costs All hospital costs are presented in billion United States dollars ($US) for the years 2010 to 2020. IBD, inflammatory bowel disease P_trend_ = Cuzick's nonparametric trend test; significance set at Pt_rend_<0.05

Variable	Billion $US											
	2010	2011	2012	2013	2014	2015	2016	2017	2018	2019	2020	Ptrend
All IBD hospitalizations	13.3	13.9	14.2	16.6	16.8	17.1	17.5	18.1	19.3	20.6	22.4	<0.001
Males	6.8	6.9	7.2	7.4	7.6	7.7	7.7	7.6	8.1	9.1	10.3	0.001
Females	6.5	7.0	7.0	9.2	9.2	9.4	9.8	10.5	11.2	11.5	12.1	<0.001
18-40 years	4.8	4.8	5.0	5.1	5.2	5.2	5.1	5.2	5.5	5.7	5.8	0.439
41–60 years	4.3	4.6	4.7	5.2	5.5	5.6	5.9	6.4	6.8	6.9	7.1	<0.001
>60 years	4.2	4.5	4.2	6.3	6.1	6.3	6.3	6.9	7.5	8.2	8.7	<0.001

Hospital Complications and Emergency Department Visits for IBD

Common complications recorded during hospitalizations for IBD were trended over the study period. Incidence of Malnutrition increased from 101,283 (30.4%) in 2010 to 104790 (30.7%) in 2020 (P<0.01). The combined incidence of bowel perforations and fistulae increased from 3,777 (1.1%) in 2010 to 4,860 (1.4%) in 2020 (P<0.001). The incidence of critical care hospitalization increased from 1,735 (0.5%) to 2,936 (0.9%) in 2020 (P<0.01; Table 5). 

**Table 5:** Trends in Hospital Complications Incidence of complications is presented as frequency (n) with proportion of total IBD hospitalization in the given year (%) IBD, inflammatory bowel disease; CCU, critical care unit P_trend_ = Cuzick's non parametric trend test; significance set at P_trend_<0.05

Complications	2010, n (%)	2011, n (%)	2012, n (%)	2013, n (%)	2014, n (%)	2015, n (%)	2016, n (%)	2017, n (%)	2018, n (%)	2019, n (%)	2020, n (%)	P_trend_
Malnutrition	101,283 (30.4)	102,634 (30.0)	102,394 (29.8)	103,404 (29.1)	104,947 (29.3)	105,352 (29.5)	105,485 (29.4)	107,305 (29.9)	108,860 (30.1)	109,362 (30.3)	104,790 (30.7)	<0.001
Anemia	37,089 (11.1)	37,112 (10.9)	37,234 (10.8)	37,847 (10.7)	38,333 (10.7)	38,029 (10.6)	38,402 (10.7)	38,460 (10.7)	38,360 (10.6)	38,880 (10.8)	38,375 (11.2)	0.0801
Bowel resection	9,864 (3.0)	9,383 (2.7)	9,937 (2.9)	10,286 (2.9)	10,372 (2.9)	10,453 (2.9)	10,536 (2.9)	10,655 (3.0)	10,385 (2.9)	10,280 (2.8)	9,869 (2.9)	0.363
Bowel perforation/fistulae	3,777 (1.1)	3,804 (1.1)	3,704 (1.1)	3,792 (1.1)	3,890 (1.1)	4,088 (1.1)	4,106 (1.1)	4,155 (1.2)	4,235 (1.2)	4,570 (1.3)	4,860 (1.4)	<0.001
Sepsis	5,106 (1.5)	5,083 (1.5)	5,102 (1.5)	5,201 (1.4)	5,189 (1.5)	5,224 (1.5)	5,305 (1.5)	5,210 (1.4)	5,200 (1.4)	5,300 (1.5)	5,335 (1.6)	0.071
CCU admissions	1,735 (0.5)	1,733 (0.5)	1,873 (0.5)	1,739 (0.5)	1,674 (0.5)	1,893 (0.5)	1,937 (0.5)	2,137 (0.6)	2,689 (0.7)	2,924 (0.8)	2,936 (0.94)	< 0.001

There were 6,243,807 ED visits for CD or UC over the study period. There was no significant trend change in overall ED visits for IBD (APC: 0.1%; CI: -0.42 to 0.54; P = 0.792). Similar outcomes were observed for males (APC: 0.1%; P = 0.452) and females (APC: 0.02%; P = 0.929). We analyzed trends in outcomes of ED visits among patients with IBD. Overall, most (55.6%) ED visits ended in routine home discharges from the ED (APC: -0.1%; CI: -1.34 to 1.13; P = 0.838), 43.3% resulted in in-hospital admissions at the same hospital (APC: 0.7%; CI: -0.10 to 2.46; P = 0.375), and 1.5% resulted in transfers to other short-term hospitals (APC: 2.9%; CI: -2.47 to 8.57; P = 0.258). A significant uptrend in ED transfers to other short-term hospitals was observed between 2016 and 2020 (APC: 17.7%; CI: 1.96-35.89; P = 0.036; Table 6).

**Table 6:** Emergency Department Visits ED, emergency department; IBD, inflammatory bowel disease P_trend_ = Cuzick's nonparametric trend test; significance level set at P_trend_<0.05

Demographic	2010, n (%)	2011, n (%)	2012, n (%)	2013, n (%)	2014, n (%)	2015, n (%)	2016, n (%)	2017, n (%)	2018, n (%)	2019	2020	P_trend_
Total ED visits related to IBD	574,038	575,866	577,948	580,463	578,587	579,745	583,472	581,027	581,177	582,837	448,647	0.368
Male	242,244 (42.2)	238,409 (41.4)	229,425 (39.7)	233,927 (40.3)	238,378 (41.2)	230,739 (39.8)	238,057 (40.8)	238,803 (41.1)	235,958 (40.6)	236,049 (40.5)	179,010 (39.9)	0.090
Female	331,794 (57.8)	337,457 (58.6)	348,523 (60.3)	346,536 (59.7)	340,209 (58.8)	349,006 (60.2)	345,415 (59.2)	342,224 (58.9)	345,219 (59.4)	346,788 (59.5)	269,637 (60.1)	0.461
ED Outcomes												
Treated and released from the ED	314,573 (54.8)	328,819 (57.1)	323,650 (56.0)	331,444 (57.1)	349,465 (60.4)	311,903 (53.8)	322,660 (55.3)	332,928 (57.3)	320,229 (55.1)	315,898 (54.2)	251,242 (56.0)	0.826
Admitted to the same hospital	249,707 (43.5)	233,802 (40.6)	246,784 (42.7)	238,570 (41.1)	209,448 (36.2)	251,030 (43.3)	238,057 (40.8)	239,383 (41.2)	251,650 (43.3)	260,528 (44.7)	187,086 (41.7)	0.183
Transferred to another short-term hospital	4,707 (0.8)	5,183 (0.9)	6,935 (1.2)	8,533 (1.5)	6,985 (1.2)	8,015 (1.4)	5,834 (1.0)	6,972 (1.2)	8,718 (1.5)	8,160 (1.4)	8,076 (1.8)	0.037

## Discussion

Our analysis of hospitalizations and ED visits for IBD in the United States from 2010 to 2020 reveals several useful insights into the evolving landscape of IBD care. Notably, the study demonstrates an overall upward trend in IBD-related hospitalizations, with a particularly marked increase in Crohn’s disease cases up to 2014, followed by a stabilization and slight decline. This increase could reflect enhanced recognition and diagnosis of Crohn’s disease by physicians or more thorough documentation of secondary diagnoses. In contrast, hospitalizations for ulcerative colitis have shown a steady increase throughout the past decade. These trends reflect broader shifts in IBD epidemiology, possibly influenced by advances in diagnostic capabilities, changes in environmental risk factors, and increased awareness of IBD symptoms among patients and healthcare providers.

The demographic analysis highlights a gradual increase in the mean age of hospitalized patients with IBD, alongside a consistent predominance of female patients over the study period. This shift toward older age groups in hospitalizations aligns with improvements in the long-term management and care transition of IBD in younger patients [13] and reflects an aging population with IBD. The predominance of hospitalizations among white Americans, followed by blacks, Hispanics, and Asians, parallels the recognized racial and ethnic disparities in IBD prevalence and healthcare access, highlighting ongoing challenges in equitable care delivery for IBD [14,15]. The study identified an upward trend in the Charlson comorbidity index (CCI) score of ≥3 among hospitalized patients with IBD, indicating an increase in the burden of comorbid conditions over time. This finding aligns with the increasing age and current understanding that comorbidities in patients with IBD, such as cardiovascular disease, diabetes, and osteoporosis, contribute to the complexity of disease management and the need for comprehensive care strategies [16]. The findings of the index study correlate with those of similar studies conducted in other settings [17].

Before 2010, trends in hospitalizations for IBD showed a steady increase, reflecting the growing prevalence and recognition of these conditions. This period was characterized by evolving diagnostic criteria and advancements in medical imaging and endoscopic techniques, which likely contributed to more accurate and earlier diagnoses. In addition, there was an increasing emphasis on the need for comprehensive care, including the use of immunomodulators and the introduction of biologic therapies, which began to change the landscape of IBD management [18,19]. Despite these advancements, the period also highlighted significant challenges, including variations in access to care, disparities in healthcare utilization among different demographic groups, and the high costs associated with hospitalizations [20]. The burden of hospitalizations was further compounded by the high rates of complications and surgery, particularly among patients with CD.

Mortality rates among hospitalized patients with IBD in the index study showed a progressive increase over time, particularly among middle-aged and older individuals. This finding can be attributed to several interrelated factors. These include the aging population with its inherent increase in comorbid conditions, heightened disease severity, and the prevalence of comorbidities such as diabetes and hypertension. In addition, evolving treatment paradigms, including the use of biologic therapies with associated risks, disparities in healthcare access, and changes in healthcare policies, may have influenced mortality rates. Socioeconomic disparities, lifestyle factors, and possibly environmental and genetic influences, along with improved diagnostic and coding practices, could also contribute to this trend [21,22]. The index study focuses on patients admitted to the hospital because of active flare-ups, disease progression, or severe manifestations of IBD and presents a unique mortality profile.

Current literature has highlighted that significant strides in the management of IBD have played a crucial role in substantially reducing mortality rates on a global scale [23]. The introduction of biologic therapiestargeting specific pathways of the immune system has markedly improved treatment outcomes [24], while therapeutic drug monitoring ensures the optimization of these treatments for individual patients [25]. Integrated care models have enhanced patient management through coordinated care, thereby addressing the comprehensive needs of patients with IBD [26,27]. In addition, advances in minimally invasive surgical techniques have minimized postoperative complications, further contributing to mortality reduction globally. Furthermore, the shift toward precision medicine has introduced genuinely personalized care for patients with IBD, ensuring that the entire patient journey, from diagnosis to treatment, is tailored to each individual’s unique biology [28]. Together, these advancements have revolutionized IBD care in the last decade.

Despite these advancements, mortality rates among hospitalized individuals with IBD have progressively increased. This paradox can be attributed to several factors. First, there is the challenge of access to and affordability of advanced treatments, which may not be universally available to all patients due to socioeconomic disparities or healthcare infrastructure limitations [29,30]. Second, the heterogeneity of IBD itself means that not all patients respond equally to treatments, with some exhibiting refractory disease despite the use of biologics or other advanced targeted therapies. In addition, the increased risk of comorbid conditions associated with IBD, such as cardiovascular disease and cancer, may not be fully mitigated by treatments that focus primarily on intestinal inflammation. There is also the impact of diagnostic delay and suboptimal monitoring, which can lead to complications or severe disease progression before effective treatment begins. Furthermore, the psychological and social burdens of living with a chronic disease such as IBD can impact overall health and well-being, potentially contributing to increased mortality. The association between female sex and lower adjusted odds of mortality is intriguing, indicating potential differences in disease severity, treatment adherence, or healthcare-seeking behavior between genders that warrant further investigation.

The analysis of healthcare utilization in this study revealed a significant increase in both the number of in-hospital procedures and total hospitalization costs over the past decade. This trend reflects not only the rising costs of medical care but also the increasing complexity of cases that reach hospitalization, necessitating more intensive diagnostic and therapeutic interventions. The disparities in hospitalization costs among racial and ethnic groups further emphasize the need to address socioeconomic and access-related factors in IBD care. ED visits for IBD remained stable over the study period, with a notable increase in transfers to other short-term hospitals from 2016 to 2020. This trend may indicate a growing reliance on specialized care centers for managing complex IBD cases or reflect broader changes in healthcare system dynamics and referral patterns.

The increasing number of hospitalizations and rising costs for IBD underscore the urgency of early, effective disease management, incorporating disease-modifying treatments, and patient education. The aging demographics of patients with IBD necessitate geriatric-focused care models tailored to their unique needs. Furthermore, disparities in hospitalization rates and costs highlight the need for culturally sensitive care and strategies to improve healthcare access for all racial and ethnic groups. The trend toward increased ED transfers calls for better care coordination and access to specialized IBD care. Ongoing research is vital to understand the complex factors driving IBD trends and to inform policy and clinical practice improvements aimed at enhancing IBD care. This study’s insights into IBD hospitalization and ED visit trends over a decade provide a crucial foundation for future efforts to optimize care and outcomes for patients with IBD, emphasizing the importance of comprehensive, integrated, and equitable care strategies.

Limitations

The index study faces limitations due to its reliance on de-identified data, which prevents the tracking of patients over time, thus constraining our understanding of long-term outcomes and quality of life. Furthermore, by focusing solely on hospitalization data, the study predominantly captures trends in more severe cases requiring hospital admission, leaving out those managed in outpatient settings. Additionally, the study exclusively utilizes data from U.S. hospitals, suggesting that caution is advised when applying its findings to countries outside the U.S., where differences in disease burden, socioeconomic factors, and geographic variations could significantly alter the results.

## Conclusions

The study observed an overall uptrend in IBD-related hospitalizations, with CD hospitalizations rising sharply until 2014, then leveling off and slightly declining, while UC hospitalizations consistently increased over the decade. Additionally, there was a noticeable rise in the average age of hospitalized IBD patients, along with a continuous female predominance throughout the study. The findings further highlight substantial disparities in hospitalization costs across different racial and ethnic groups, including Black, Hispanic, and Asian/Pacific Islander individuals, as well as among women, middle-aged individuals, and the elderly. Existing data indicate that minority populations, including Black, Hispanic, and Asian/Pacific Islander individuals, often face barriers to timely access to care (including lack of insurance coverage, low income levels, and geographic variations in care), leading to more severe disease presentations and higher resource utilization during hospitalizations. Systemic inequities in healthcare delivery further exacerbate these disparities. This emphasizes the need to address these inequities to ensure equitable care for all IBD patients. Overall, there has been a growing trend in comorbidity burden, resource use, and in-hospital mortality among IBD patients, especially in the middle-aged and elderly demographics.
